# Application of Subperineural Resection Technique in Vestibular Schwannomas: Surgical Efficacy and Outcomes in 124 patients

**DOI:** 10.3389/fonc.2022.849109

**Published:** 2022-04-20

**Authors:** Yingxi Wu, Chen Wei, Ping Wang, Yunze Zhang, Yang Wu, Yafei Xue, Tianzhi Zhao, Yan Qu

**Affiliations:** Department of Neurosurgery, Tangdu Hospital, The Air Force Medical University, Xi’an, China

**Keywords:** subperineural resection technique, facial–acoustic nerve function, gross total resection, neurophysiological monitoring, vestibular schwannomas, microsurgery, extent of tumor adhesion

## Abstract

**Objective:**

We aimed to explore the application and prospects of the subperineural resection technique for tumor separation and removal under the perineurium during surgery for vestibular schwannomas (VSs).

**Methods:**

This study retrospectively analyzed 124 patients with VSs who underwent surgery *via* a retrosigmoid approach from July 2015 to October 2020 in the Department of Neurosurgery, Tangdu Hospital, Air Force Military Medical University. The data will be discussed with regard to the following aspects: clinical features, surgical strategies, tumor resection extent, facial–acoustic function preservation, and postoperative complications.

**Results:**

Gross total resection (GTR) of the tumor was achieved in 104 patients, with a GTR rate of 83.9%, and subtotal resection (STR) of the tumor was achieved in 20 patients. There was no significant difference in facial and acoustic nerve functional preservation between GTR and STR, as well as in tumor resection between solid and cystic tumors. The retention rate reached 97.6% in terms of complete anatomical facial nerve preservation. Facial nerve function was assessed using the House–Brackmann (HB) grading score. Consequently, HB grades of I–II, III–IV, and V–VI were determined for 96 (77.4%), 25 (20.2%), and 3 (2.4%) cases, respectively, 1 week postoperatively and accounted for 110 cases (88.7%), 13 cases (10.5%), and 1 case (0.8%), respectively, at 6 months. Fifteen of 35 (42.9%) patients with serviceable hearing before the operation still had serviceable hearing at 6 months postoperatively. There were 5 cases of cerebellar or brainstem bleeding after the operation, and one patient died. Multivariate logistic regression analysis showed that older age (≥60 years, *p* = 0.011), large tumor (>3 cm, *p* = 0.004), and cystic tumor (*p* = 0.046) were independent risk factors associated with the extent of adhesion between the tumor and the brainstem and facial–acoustic nerve.

**Conclusion:**

We successfully applied the subperineural resection technique to a large series of patients with VSs and achieved satisfactory results. Accurate identification of the perineurium and subperineural resection of the tumor can effectively reduce the disturbance of the facial–acoustic nerve during the operation and provide an intuitive basis for judging the tumor boundary. The subperineural resection technique may be conducive to improving the rate of total tumor resection and facial–acoustic nerve functional preservation in the surgical treatment of VSs.

## Introduction

With the development of microneurosurgical techniques, the application of various advanced instruments, and intraoperative electrical nerve monitoring (1, 2), the gross total resection (GTR) rate and the postoperative preservation of facial nerve function in vestibular schwannomas (VSs) have significantly improved in recent years. Meanwhile, hearing retention is increasingly becoming the goal of surgical treatment (3, 4). According to previously reported results, in patients with small tumors (<1.5 cm in diameter), 40%–70% have serviceable hearing postoperatively and less than 10% of these have permanent facial weakness (5–7). However, for patients with tumors larger than 2.5 cm in diameter, the rate of serviceable hearing preservation after surgery is less than 5%, and the risk of partial or permanent facial paralysis is about 50% in those with total resection of large tumors (8–10). The complexity of the structure increases with tumor volume, which may cause difficulties for neurosurgeons to identify and protect cranial nerves (CNs) VII/VIII and the brainstem. Surgical techniques based on a better understanding of the membranous characteristics of VSs are necessary for the achievement of satisfactory results.

Sasaki et al. (11) firstly demonstrated the perineurium structure around VSs based on histological observations and introduced the subperineural dissection technique in surgery. Satisfactory resection rates and facial nerve function preservation were achieved in subsequent reports of the application of this technique in VS treatment (12, 13). However, the complicated membranous structure around VSs makes understanding and duplicating the subperineural separation technique difficult. Moreover, there is no report on the effects of the application of this technology in a large series of cases so far. In this study, we applied the subperineural resection technique in the surgical treatment of 124 patients with VSs. We proposed an easily accessible model of membranous structure around VSs and introduced the nuances and pearls of the subperineural technique based on this model in protecting related cranial nerves and the brainstem. Furthermore, we retrospectively analyzed the surgical effects and complications of this technique and presented our experiences.

## Methods

### Baseline Characteristics of the Patients

A total of 124 patients with VSs recruited from July 2015 to October 2020 underwent microsurgical treatment in the Department of Neurosurgery, Tangdu Hospital, Air Force Military Medical University. Among these patients, 48 were men and 76 were women. The age range of the patients was 21–75 years, with an average age of 48.5 ± 12.9 years (**Table 1**). VSs were confirmed pathologically in all patients. Patients diagnosed with neurofibromatosis type II and who previously received surgical treatment were excluded.

**Table 1:** Clinical features of 124 patients with vestibular schwannoma.

Items	Values (%)
Total cases	124
Male	48 (38.7)
Female	76 (61.3)
Mean age	48.5 (21–75)
Mean diameter of tumor (cm)	3.0 (0.6–5.5)
≤3	71 (57.3)
>3	53 (42.7)
Tumor texture	
Cystic	32 (25.8)
Solid	92 (74.2)
Tumor grade (Samii)	
T1	2 (0.16)
T2	8 (6.5)
T3	43 (34.7)
T4	71 (57.3)
Clinical symptoms	
Hearing impairment	85 (68.5)
Tinnitus	38 (30.6)
Facial numbness or hypoesthesia	34 (27.4)
Facial pain	2 (1.6)
Facial paralysis	9 (7.3)
Unsteady walking	10 (8.1)
Headache and dizziness	37 (29.8)
Hoarse voice and choking cough	2 (1.6)
Vertigo	11 (8.9)
Tumor resection	
Total resection	104 (83.9)
Subtotal removal	20 (16.1)
Preoperative facial nerve function	
Normal (I–II)	122 (98.4)
Moderate decline (III–IV)	1 (0.8)
Severe decline (V–VI)	1 (0.8)
Postoperative facial nerve retention	
Anatomical preservation	121 (97.6)
Functional preservation (1 week later)	
Normal (I–II)	96 (77.4)
Moderate decline (III–IV)	24 (20.2)
Severe decline (V–VI)	4 (2.4)
Functional preservation (6 months later)	
Normal (I–II)	110 (88.7)
Moderate decline (III–IV)	13 (10.5)
Severe decline (V–VI)	1 (0.8)
Preoperative hearing	
Serviceable hearing	35 (28.2)
Unserviceable hearing	89 (71.8)
Postoperative hearing (6 months later)	
Serviceable hearing	15/35 (42.9)
Unserviceable hearing	20/35 (57.1)

### Preoperative and Postoperative Radiological Examination

Preoperative hearing assessment included pure tone hearing, speech discrimination score, and acoustic brainstem response. The following House–Brackmann (HB) grading system was used to evaluate facial nerve function (14): grades I–II, good function; grades III–IV, moderate decline; and grades V–VI, severe decline. Facial nerve function was assessed on the first day and at 1 week, 3 months, 6 months, and 1 year postoperatively. Magnetic resonance imaging (MRI) plain and enhanced scans were performed in all patients before the operation and at 3 days, 3 months, 6 months, and 1 year postoperatively, and then annually at follow-up visits. Samii classification was utilized to assess tumor size and compression on the surrounding cerebellum and brainstem (15). Thin-layer CT scan of the petrous bone was performed to observe the size of the internal auditory canal and the location of the labyrinth; moreover, the presence of a high jugular bulb was detected.

### Intraoperative Electrophysiological Monitoring

For the facial nerve, the recording electrodes were placed on the ipsilateral orbicularis oculi and orbicularis oris. For the trigeminal nerve, the electrode was placed on the ipsilateral masticatory muscles. For the accessory nerve, the electrode was placed on the ipsilateral trapezius muscle or sternocleidomastoid muscle. For the auditory nerve, a positive electrode on the top of the head and negative electrodes on both sides of the auricular lobule were able to monitor brainstem auditory-evoked potentials. The stimulation electrode was located in the internal auditory canal.

### Surgical Method

**Figure 1:**
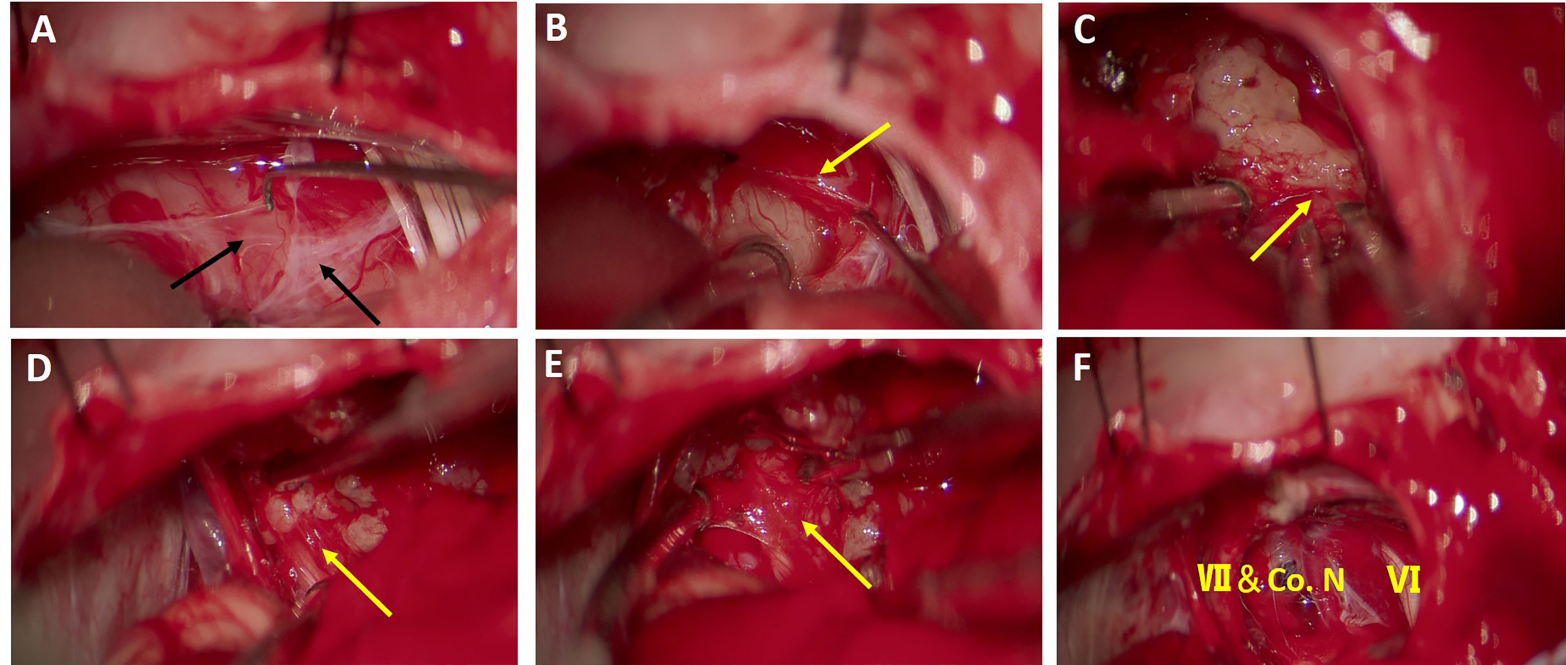
Surgical procedure of tumor separation and removal under the perineurium. **(A)** An arachnoid fold consisting of the first and second layers of the arachnoid membrane can be identified on the superficial surface of the tumor. **(B)** Under the arachnoid fold, the tumor capsule was identified and reflected under the perineural plane to create a distinguished interface for resection of the tumor. **(C)** The tumor was separated from the surface of the brainstem, with the perineurium remaining on the brainstem. **(D)** Rostrally, facial nerve was preserved under the perineurium and the tumor was dissected from the perineurium. **(E)** The perineurium above the facial nerve and cochlear nerve was kept intact after tumor removal. **(F)** Cerebellopontine angle (CPA) area after tumor removal. The brainstem, facial nerve (VII), cochlea nerve (*Co. N*), and abducens nerve (VI) were preserved well after subperineural tumor resection. *Black arrows* indicate arachnoid membrane and *yellow arrows* indicate the perineurium.

**Figure 2:**
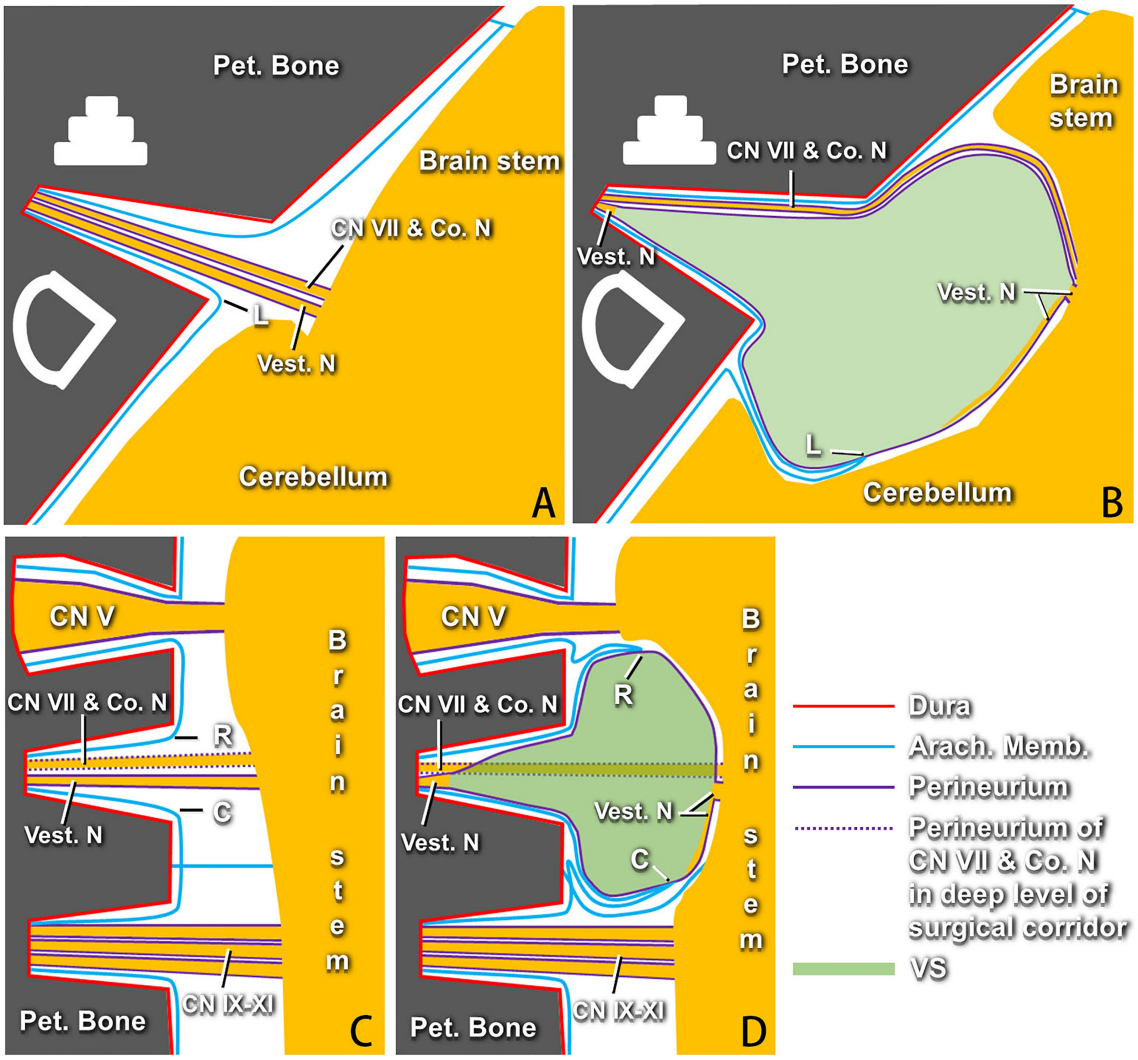
Models of membranous structures around vestibular schwannomas (VSs) proposed by the authors according to the surgical findings (*right side*). **(A)** Axial section showing the facial nerve, cochlear nerve, and vestibular nerves, which are covered with perineurium, running from the brainstem into the internal acoustic canal (IAC) fundus under the arachnid membrane in the normal situation. Point “*L*” indicates the arachnoid membrane at the lateral point of the IAC porus. **(B)** Axial section showing the arachnoid membrane at point “*L*” being adhered and stretched by the tumor to the cerebellopontine angle (CPA) with the progress of VSs, which causes “three arachnoid layers” on the lateral surface of the perineurium-covered tumor anterior to the dislocated point “*L*.” The site of point “*L*” pulled by the tumor to the CPA is different for each case. There remain some degenerated vestibular nerve fibers that can be identified between the perineurium and the tumor, as well as the IAC fundus. **(C)** Coronal section showing the normal profile of membranous structures at the rostral and caudal sides of cranial nerves (CNs) VII and VIII. Points “*R*” and “*C*” represent the arachnoid membrane at the rostral and caudal points of the IAC porus, respectively. **(D)** Membranous structures between the VSs and CN V or CN IX–XI varying greatly at different sites due to the different dislocations of points “*R*” and “*C*.” The innermost layer of the perineurium can be identified constantly around the tumor. *Pet*., petrous; *Co. N*, cochlear nerve; *Vest. N*, vestibular nerve; *Arach*., arachnoid; *Memb*., membrane; *VSs*, vestibular schwannomas.

Lumbar cistern drainage was performed routinely before surgery. A craniotomy was conducted with a suboccipital retrosigmoid approach in the lateral prone position intraoperatively (16). The transverse sinuses and the intersection of transverse and sigmoid were located on the basis of MRI. The bone flap was designed according to tumor size. Cerebrospinal fluid (60–80 ml) was released after bone removal. When the cerebellum collapses naturally, the arachnoid fold (the first and second layers of the arachnoid) (**Figures 1A**, **2B**) can be discerned with separation along the cerebellopontine angle (CPA) cistern. The deep tumor and its surface “capsule” (the third layer of the arachnoid membrane plus the perineurium anterior to point L, or the perineurium only posterior to point L) (**Figures 1A**, **2B**) can be seen when the arachnoid fold is pulled apart. Electrical stimulation on the surface of the tumor was used to detect the facial nerve. In the position where the facial nerve is not detected, the “capsule” of the tumor was separated from the surface of the tumor using a hook microdissector under the perineural layer (**Figures 1B**, **2B**), and the tumor was removed according to the order: center, brainstem end (**Figures 1C**, **2B**), caudal end, and rostral end (**Figures 1D**, **2D**). As shown in **Figure 2**, even the “capsule” of the tumor consists of different layers of membranous structure at different points individually, and the perineurium is always the innermost and only layer that fully covers the tumor. Identifying the interface between the tumor and the perineurium and keeping resection of the tumor under the perineurium (subperineural technique) are vital to guarantee that the brainstem is intact and CN VII and the cochlear nerve are out of the tumor “capsule” (**Figures 1E, F** **)**. A blunt or sharp separation technique was applied according to the degree of adhesion of the tumor to the perineurium.

After removing the CPA tumor, individualized drilling of the lateral wall of the internal acoustic canal (IAC) was performed. Over-drilling was avoided by continuous checking in order to protect hearing structures until the outermost pole of the tumor can just be directly seen with opening of the dura. The superficial layers of the arachnoid membrane and the perineurium on the IAC tumor surface were opened (**Figure 2D**). After debulking, the tumor in the IAC was also separated bluntly or sharply from the perineurium and dissected backward from the fundus to the porus of the IAC. When the tumor was resected completely, bone wax and muscle were applied to close the opened lateral wall of the IAC, the layers were sutured, and the incision was closed after hemostasis.

### Definition of Extent on Tumor Adhesion

Mild adhesion: the tumor can be easily detached from the perineurium with a “peeling-off” technique.

Moderate adhesion: the tumor cannot be detached directly from the perineurium. A microdissector is required to create an interface between the tumor and the perineurium for further subperineural resection.

Severe adhesion: even with manipulation using the microdissector, the tumor cannot be separated from the perineurium. A sharp dissection technique is required in this situation.

### Statistical Analysis

SPSS 23.0 was used for statistical analysis. The measurement data were expressed as *x* ± *s* with a *t*-test, and the enumeration data were expressed as *n* (percent) with a chi-squared test or Fisher’s exact test. Univariate analysis was performed to assess influencing factors associated with the extent of tumor adhesion. Multivariate logistic regression was then performed to identify the independent predictors found significant in the univariate analysis. A *p*-value <0.05 was considered as statistically significant.

## Outcomes

### Extent of Tumor Resection

**Figure 3:**
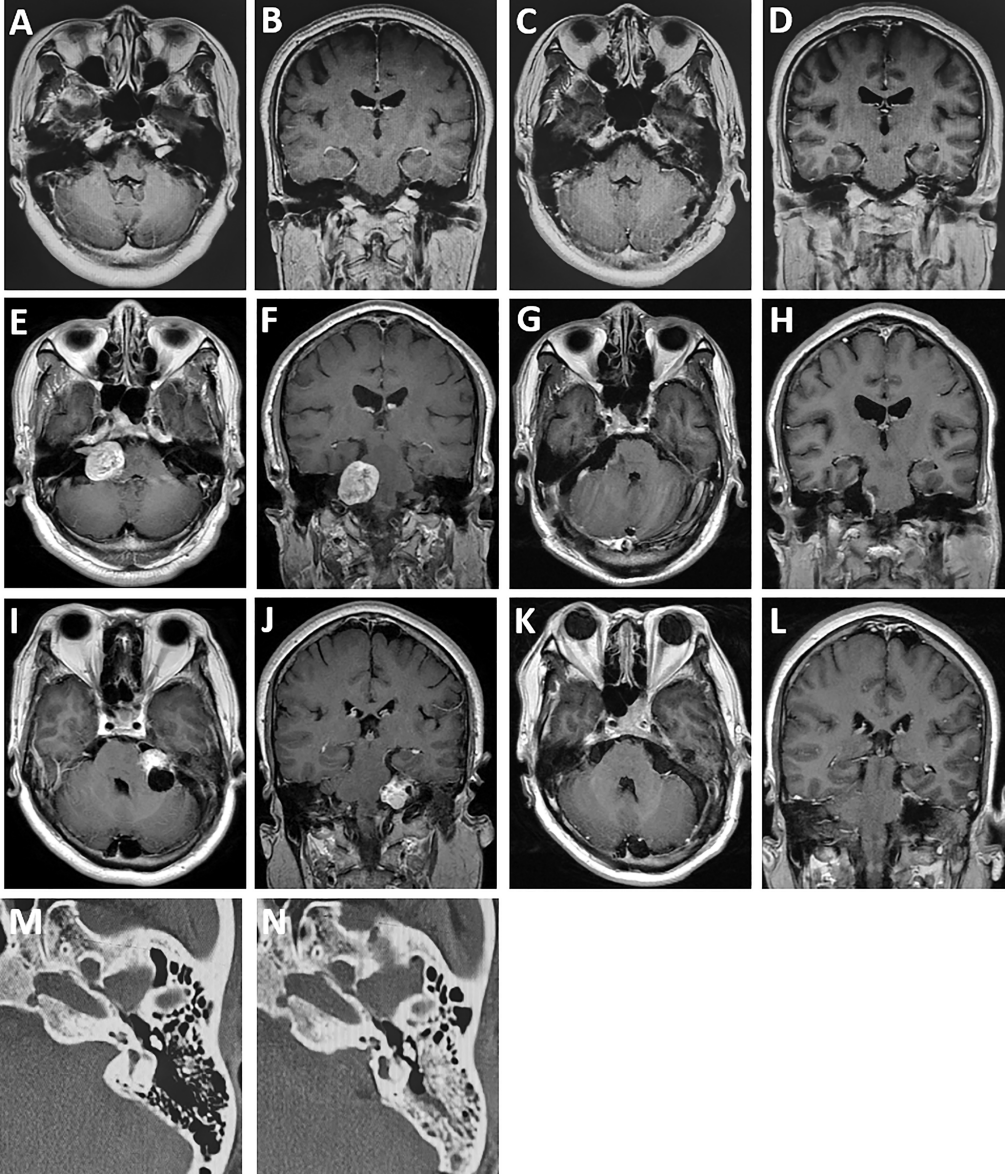
Comparison of preoperative and postoperative MRI. **(A, B)** A patient with vestibular schwannoma in the internal auditory canal with effective hearing preoperatively. **(C, D)** MRI at 6-month follow-up revealing total tumor resection and hearing preservation obtained in the patient. **(E, F)** A patient with solid vestibular schwannoma in the cerebellopontine angle. **(G, H)** Baseline MRI at 6-month follow-up revealing gross total resection of the tumor and good facial nerve functional preservation (House–Brackmann grade I) after surgery. **(I, J)** A patient with predominantly cystic vestibular schwannoma with hearing loss before surgery. **(K**, **L)** MRI at 6-month follow-up showing no tumor recurrence. **(M**, **N)** Comparison of the bone window CT scans of the internal auditory canal before and after grinding.

The extent of tumor resection was distinguished by postoperative MRI performed 3 months after surgery (**Figure 3**). Among the 124 patients with VSs, 104 had GTR, with a GTR rate of 83.9%, and 20 underwent STR, with an STR rate of 16.1%. There was no significant difference in facial and acoustic nerve functional preservation and in the configuration of the facial nerve in relation to the tumor between GTR and STR (**Table 2**). No significant difference was observed in the tumor resection between solid and cystic tumors (**Table 2**).

**Table 2:** Analysis of categorical variables between the gross total resection (GTR) group and subtotal resection (STR) group.

Parameter	GTR group	STR group	*p*-value
No. of patients	104	20	
Tumor texture			0.93
Solid, *n* (%)	77	15	
Cystic, *n* (%)	27	5	
Facial function preservation			0.55
Good, *n* (%)	79	17	
Decline, *n* (%)	25	3	
Hearing preservation			0.70
Effective hearing, *n* (%)	11	4	
Invalid hearing, *n* (%)	16	4	
Configuration of facial nerve in relation to tumor			1.0
Ventral side of tumor, *n* (%)	69	14	
Upper pole of tumor, *n* (%)	21	4	
Dorsal of tumor, *n* (%)	1	0	
Lower pole of tumor, *n* (%)	13	2	

### Facial Nerve Anatomical and Functional Preservation

The facial nerve was completely anatomically preserved after the operation in 121 cases (97.6%); the facial nerve was not completely retained in the other 3 because of tumor erosion or adhesion. The HB scores for facial nerve function 1 week after the operation were as follows: 96 cases (77.4%) were of grades I–II (good function), 25 cases (20.2%) of grades III–IV (moderate decline), and 3 cases (2.4%) of grades V–VI (severe decline). The scores at 6 months after operation were as follows: 110 cases (88.7%) with grades I–II, 13 cases (10.5%) with grades III–IV, and 1 case (0.8%) with grade V–VI.

### Hearing Preservation

Among 35 patients with serviceable hearing before the operation, 17 had normal hearing and 18 patients had a hearing decline. At 6 months post-operation, of the 17 patients with normal hearing preoperatively, 3 (17.6%) still had normal hearing postoperatively, 5 (29.4%) had a hearing decline, and 9 (52.9%) had hearing loss. Of the 18 patients with a hearing decline, 7 still had serviceable hearing and the other 8 had hearing loss. Thus, 15 out of 35 (42.9%) patients still had serviceable hearing 6 months after the operation.

### Predictors for Postoperative Hearing Preservation

Some potential predictors including the preoperative hearing level, origin of the tumor, tumor texture, maximum diameter of the tumor, fundal fluid, and adhesion were analyzed. The results demonstrated that there was better hearing preservation with fundal fluid on preoperative MRI than without fundal fluid. The hearing preservation rate (4/5, 80%) with maximum tumor diameter ≤2 cm was higher than that (11/30, 37%) with maximum tumor diameter >2cm, although there was no significant statistical difference (**Table 3**).

**Table 3:** Predictors affecting postoperative hearing preservation.

**Variable**	Postoperative hearing status		*p*-value
	Serviceable hearing	Unserviceable hearing
Preoperative hearing level (AAO-HNS Scale)			0.74
A	8	9	
B	7	11	
Origin of tumor			0.82
Superior vestibular nerve	5	5	
Inferior vestibular nerve	3	5	
Non-identifiable	7	10	
Tumor texture			1
Cystic	5	6	
Solid	10	14	
Maximum diameter of tumor (cm)			0.14
≤2	4	1	
>2	11	19	
Fundal fluid			0.03
Yes	9	4	
No	6	16	
Adhesion			0.67
Weak or intermediate	13	15	
Strong	2	5	

AAO-HNS, American Academy of Otolaryngology–Head and Neck Surgery Classification.

### Postoperative Radiotherapy

All patients with GTR of VSs had not received radiotherapy. Thirteen of the 20 patients undergoing STR of lesions received stereotactic radiosurgery 1 month after surgery. Contrast-enhanced T1- and T2-weighted (T2W) MRI, as well as contrast-enhanced CT scans, was used to plan the radiosurgery. A prescription dose of 12 Gy was delivered to the planning target volume for the tumor.

### Risk Factors Related to Extent of Tumor Adhesion

We observed that the extent of adhesion of the tumor to the brainstem and facial–acoustic nerve during surgery was the important risk factor that affected the total resection rate of tumor and the preservation rate of facial and acoustic nerve function; therefore, candidate variables including sex, age, maximum diameter of tumor, tumor texture, Ki-67, peritumoral edema, Samii classification, and tumor blood supply were analyzed. The outcomes revealed that age (*p* = 0.0046), maximum diameter of tumor (*p* = 0.0023), tumor texture (*p* = 0.033), and peritumoral edema (*p* = 0.045) significantly affected the extent of tumor adhesion (**Table 4**). When age, maximum diameter of tumor, tumor texture, and peritumoral edema were entered into the multivariate logistic regression analysis, older age (≥60 years, *p* = 0.011), large tumor (>3 cm, *p* = 0.004), and cystic tumor (*p* = 0.046) were independent risk factors for the extent of tumor adhesion (**Table 5**).

**Table 4:** Statistical analysis for potential risk factors related to extent of tumor adhesion.

Variable	Extent of tumor adhesion		*p*-value
	Weak or intermediate	Strong	
Age (years)			0.0046
<60	76 (76.8)	23 (23.2)	
≥60	12 (48)	13 (52)	
Sex			0.40
Male	32 (66.7)	16 (33.3)	
Female	56 (73.7)	20 (26.3)	
Maximum diameter of tumor (cm)			0.0023
>3	30 (56.6)	23 (43.4)	
≤3	58 (81.7)	13 (18.3)	
Tumor texture			0.033
Cystic	18 (56.2)	14 (43.8)	
Solid	70 (76.1)	22 (23.9)	
Ki-67			0.12
<3%	64 (75.3)	21 (24.7)	
≥3%	24 (61.5)	15 (38.5)	
Peritumoral edema			0.045
No	85 (73.3)	31 (26.7)	
Yes	3 (37.5)	5 (62.5)	
Samii classification			0.28
I-II	9 (90)	1 (10)	
III–IV	79 (69.3)	35 (30.7)	
Tumor blood supply			0.19
Low–moderate vascular	85 (72.6)	32 (27.4)	
Hypervascular	3 (42.9)	4 (57.1)	

**Table 5:** Multivariate logistic regression for extent of tumor adhesion.

Variable	Multivariate analysis		
	OR	95% CI	*p*-value
Age (≥60 years)	3.994	1.370−11.644	0.011
Maximum diameter of tumor (>3 cm)	4.142	1.560−10.995	0.004
Tumor texture (solid)	0.360	0.132−0.981	0.046

### Procedure-Related Complications

Five cases of cerebellar or brainstem hemorrhage occurred in the operation area, of which 1 case died. Two patients underwent sustained craniotomy and decompressive craniectomy, and only mild neurological dysfunction remained postoperatively. Two patients were cured and discharged with conservative treatment. Nine patients with postoperative high fever complicated with intracranial infection were cured when they were treated with lumbar cistern drainage of the cerebrospinal fluid and anti-infection. Cerebrospinal fluid leakage occurred in 4 patients, of which 1 case was otorrhea and was cured conservatively. The remaining 3 cases developed incision leakage due to improper dura mater suture, but the leak disappeared when repaired. Cerebellar ataxia appeared postoperatively in 8 patients, but all returned to normal 3–6 months later. Symptoms of posterior cranial nerve injury emerged in 4 patients, and abducent nerve injury emerged in 2 patients postoperatively, which manifested as attenuation of the cough reflex and dysphagia and ocular movement disorder.

### Follow-Up

The follow-up period ranged from 11 to 70 months, with an average of 34.6 ± 17.4 months. During the follow-up, two patients receiving STR but no radiotherapy underwent reoperation due to tumor recurrence. The recurrence times were 28 and 41 months, respectively. The other patients had no recurrence in the follow-up period.

## Discussion

Conservative treatment, stereotactic radiotherapy, and surgery were employed in the treatment of VSs. For patients with large VSs and obvious clinical symptoms, surgery is still the only option to alleviate the clinical symptoms and cure the tumors (17). The difficulty in obtaining GTR and functional preservation of the facial and auditory nerves increases with the increase of tumor volume.

At present, studies have confirmed that most VSs are subarachnoid, whereas those located outside the arachnoid region occur quite rarely. Kohno et al. (18) confirmed that 86 of 118 patients (73%) were subarachnoid, 2 (2%) cases were extra-arachnoid, and the remaining 30 cases (25%) were relatively large tumors that were difficult to determine. Lescanne et al. (19, 20) also reported that the arachnoid membrane of the CPA cistern continued from the brainstem to the bottom of the internal carotid artery (ICA), and the facial–acoustic nerve complex had no interval and was located under the arachnoid, indicating that the VS was a non-extraarachnoid tumor. It was suggested, by means of optical microscopy and immunohistochemical staining, that the membranous structure covering the tumor surface was arachnoid (18, 21, 22). Ohata et al. (22) further elucidated the rearrangement of the adjacent arachnoid membrane with the tumor growth, which caused different capsule layers of the arachnoid membrane at different sites on VSs, and advocated that the tumor should be dissected and resected along the subarachnoid plane. Furthermore, Sasaki et al. (11) demonstrated that there is a thin layer of connective tissue membrane composed of the perineurium and that some degenerated nerve fibers under it lie on the surface of the tumor. Subsequently, the subperineural VS resection technique was introduced and demonstrated to obtain good surgical results (12, 13). However, it is still difficult to identify the perineurium accurately and to get to the right dissection plane due to the “rearrangement of adjacent arachnoid membrane” out of the perineurium, which may cause this technique to be less repeatable. Based on our surgical experience on 124 VS cases, we combined the “arachnoid membrane rearrangement theory” and the “subperineural resection technique” and proposed a more comprehensive descriptive model of the membranous structure around VSs (**Figures 2A–D**). Better understanding of the membranous structure around VSs based on this model will be greatly helpful for neurosurgeons to identify the accurate subperineural dissecting plane.

In the present study, the subperineural resection technique was demonstrated to effectively reduce the disturbance of the facial–acoustic nerve during operation and to provide an intuitive basis for judging the tumor boundary. It is important to try to avoid breaking through the perineural layer during the entire process of tumor resection. If the perineurium has been accidentally pierced and enters the extra-perineural space as a result, it is necessary to try to reenter the subperineural plane. Esquia-Medina et al. (23) also found that the degree of tumor adhesion, which is mainly evaluated by the nerve displacement, was an important predictive factor associated with facial function. In this study, we proposed a manipulation-based classification in depicting the extent of tumor adhesion to the perineurium and found that older patients and large and cystic tumors were closely associated with severe tumor adhesion, which may significantly affect total tumor removal and nerve function preservation when using this technique. When the tumor has a clear boundary with the perineurium above the brainstem and the facial and cochlear nerves, the tumor could be separated and removed under the perineurium to achieve GTR. However, in the instance of severe adhesion of the tumor to the perineurium (usually at the site near the ICA porus), the function of the facial–acoustic nerve might be damaged if the tumor and the perineurium were detached roughly from the nerves. Therefore, when the interface between the tumor and the perineurium could not be separated using the microdissector, neurosurgeons must use a microscissor to perform sharp dissection in order to create an interface at the mixed level of nerve fibers and tumor cells. In this way, although a small part of the tumor tissue may remain on the perineurium above nerves, the possibility of preserving the function of the facial–auditory nerve will be significantly improved (11, 24, 25).

In addition to the membrane resection technique, which improves the functional preservation of the facial auditory nerve, intraoperative neurophysiological monitoring also plays an important role in anatomical recognition, intraoperative functional preservation, and postoperative prediction (26, 27). Neff et al. (28) pointed out that if the stimulus threshold was ≤0.05 mA or the response amplitude was ≥240 μV, then there is a 98% chance for predicting postoperative facial auditory nerve function as HB grade I or II. Boublata et al. (29) reported on 151 cases of VSs with diameters of 31–60 mm under neurophysiological monitoring and demonstrated that the GTR rate was 82.8%, the anatomical retention rate of facial nerves was 98.7%, and that patients with HB grades of I and II at 2 years postoperatively accounted for 82%. Although the facial–acoustic nerve cannot be seen directly with neurophysiological monitoring, the relative distance between the nerve and the probe can be inferred by adjusting the current required to stimulate the muscle. This suggests that the nerve is highly exposed close to the probe when the evoked potential is obtained with a current of <0.2 mA for stimulation. In contrast, it indicated that a larger tissue or bone barrier existed between the probe and the nerve with stimulation under a current >0.5 mA (30). The retention of auditory nerve function was still not comparable to that of facial nerve function despite the combination of brainstem auditory-evoked potentials (BAEPs), electrocochleograms (ECochGs), and cochlear nerve action potentials (CNAPs) for vestibulocochlear nerve monitoring. More importantly, the larger the tumor, the higher the probability of postoperative hearing loss; for this reason, further clinical research and other electrophysiological monitoring techniques are needed to improve hearing retention (30).

Boublata et al. (29) reported on 126 patients (82.8%) who had GTR tumors, 20 patients (13.9%) who had subtotal tumors, and 124 patients (82%) who had facial nerve function of HB grades I–II 2 years after surgery. The study of Sughrue et al. (31) included 74% of patients who underwent GTR, 11.5% underwent near-total resection, and 14.5% underwent SRT. In a retrospective study of the efficacy of the facial nerve-sparing approach, functional preservation of the facial nerve with HB grade I or II was achieved in 97% after surgery (32). In our group of 124 patients with VSs, the GTR rate was 83.9%, and patients with HB grades I–II postoperatively accounted for 77.4% at 1 week and 88.7% at 6 months. These data indicated that the subperineural technique is effective in obtaining total tumor resection and in preserving facial nerve function.

Although satisfactory results were reported for the resection of total tumor and preservation of facial nerve function, preservation of hearing was still unsatisfactory, especially for large VSs. Zhu et al. (33) reported a postoperative rate of serviceable hearing preservation of 60.9% (67/110) in 110 small VSs (≤1.5cm). Wanibuchi et al. (4) also determined a hearing preservation rate of 53.7% in 592 consecutive patients with VSs (>2 cm in diameter). Surgical tips were introduced as vital to preserving hearing, including bloodless microdissection, subperineural or subcapsular dissection, real-time neurophysiological monitoring, sharp resection of the facial–acoustic nerve, and avoidance of mechanical and heat damage to the brainstem, the nerves, and the internal auditory artery (4, 33). In this study, the hearing preservation rate at 6 months was 43% (15/35). The prognostic factors were analyzed for postoperative hearing preservation, and it indicated that the presence of fundal fluid was a positive prognostic factor. Several documents also stated that the presence of fundal fluid on T2W MRI was predictive of postoperative hearing preservation (33–35). A number of studies confirmed a higher postoperative rate of hearing preservation in tumors originating from the superior vestibular nerve than in those originating from the inferior vestibular nerve, as well as in small tumors than in large tumors (33, 35). However, in our study, we did not find hearing preservation to be related to the tumor origin and size. This may be due to the limited sample of patients with serviceable hearing before operation and most of the recruited patients with preoperative serviceable hearing having large tumors, which may have resulted in poor hearing preservation rate.

The effect of stereotactic radiotherapy in VSs is more and more accepted. For small and medium VSs (≤2.8 cm), gamma knife surgery (GKS) showed good results in terms of tumor control and functional preservation of the facial and acoustic nerves (36). Larger VSs adhere more tightly to the brainstem and facial–acoustic nerve, and the risk of brainstem injury or facial paralysis may be higher after surgery. The treatment strategy combining STR with GKS has been usually selected for larger VSs, also achieving good facial–acoustic nerve functional preservation (32, 37). The classification criterion for the degree of tumor adhesion proposed in this study provides a good reference for neurosurgeons in planning surgical strategy. GTR is relatively easy to achieve in the mild and moderate adhesion group. We strongly recommend that STR combined with postoperative GKS strategy should be adopted when operating on tumors firmly adhered to the perineurium in order to maintain good life quality of patients.

The most serious complications after microsurgery of VSs were intraoperative brainstem injury and postoperative bleeding in the operation area, which were consistent with previous reports (38–40). Bleeding occurred in 5 cases. Of these patients, 1 died due to respiratory and cardiac arrest the next day postoperatively. Craniotomy and hematoma evacuation occurred in 2 cases, and conservative treatment was given in the other 2 cases; eventually, all patients were cured and discharged. Adhesion of the tumor to the brainstem and cerebellum leads to microdamage to the small blood vessels at the junction of tumor and brain tissue, which is the main cause of bleeding (40). Due to the complexity of the surgical procedures, the operation generally lasts longer, especially for larger VSs, and the risks of postoperative fever and intracranial infection could be greater. We carried out procedures such as preoperative lumbar cistern to facilitate intraoperative cerebellar collapse in order to extend the operating space and postoperative continuous drainage of the cerebrospinal fluid until it tested normal in laboratory analysis, which significantly reduced the duration of fever or infection in patients. Another common complication is cerebrospinal fluid otorrhea or incision leakage (5, 41). The incidence of leakage was 3.2% in these cases, which was not very high compared to that in previous literature (40, 41). It is recommended that once mastoid pneumatization is opened, bone wax should be used to seal it tightly, and sometimes it is necessary to cover the muscles on the mastoid for fixation. For the dura mater, a 4–0 absorbable suture was used to close the dural gap, and autologous fascia was responsible for repairing larger dura defects. The procedures were finished by covering the artificial dura, fixing with tissue colloid, and using an elastic bandage for incision compression.

## Conclusion

The membranous structure around VSs is complicated and is divergent for each case. The perineurium provides a safe and constant dissecting plane for neurosurgeons to remove the tumor. Fine outcomes have been achieved regarding the GTR rate and the facial–acoustic nerve functional preservation rate with the use of the subperineural technique under neuroelectrophysiological monitoring. The decision to perform radical GTR or STR combined with GKS can be made according to the degree of tumor adherence.

## Data Availability Statement

The raw data supporting the conclusions of this article will be made available by the authors, without undue reservation.

## Ethics Statement

Ethical review and approval was not required for the study on human participants in accordance with the local legislation and institutional requirements. Written informed consent for participation was not required for this study in accordance with the national legislation and the institutional requirements.

## Author Contributions

QY and ZT: chief surgeon, writing—review and editing. WYX, WC, and ZY: writing—original draft preparation. WP, WY, and XYF: data collection and analysis. All authors contributed to the article and approved the submitted version.

## Funding

This work was supported by the National Natural Science Foundation of China (no. 81971153) and Tangdu Hospital New Technology Plan (nos. XJSXYW2021120, XJSXYW202126).

## Conflict of Interest

The authors declare that the research was conducted in the absence of any commercial or financial relationships that could be construed as a potential conflict of interest.

## Publisher’s Note

All claims expressed in this article are solely those of the authors and do not necessarily represent those of their affiliated organizations, or those of the publisher, the editors and the reviewers. Any product that may be evaluated in this article, or claim that may be made by its manufacturer, is not guaranteed or endorsed by the publisher.

## Abbreviations

MRI, magnetic resonance imaging; CPA, cerebellopontine angle; IAC, internal auditory canal; GTR, gross total resection; STR, subtotal resection; HB, House Brackmann; GKS, gamma knife surgery; VSs, vestibular schwannomas.
